# A new genus of Grapholitini from Africa related to *Thaumatotibia* (Lepidoptera, Tortricidae)

**DOI:** 10.3897/zookeys.438.7490

**Published:** 2014-09-01

**Authors:** Alicia E. Timm, John W. Brown

**Affiliations:** 1 Department of Zoology and Entomology, Rhodes University, P.O. Box 94, Grahamstown 6140, South Africa; 2 Systematic Entomology Laboratory, ARS, USDA, National Museum of Natural History, Washington, DC 20013-7012, USA

**Keywords:** Democratic Republic of Congo, Ethiopia, Kenya, male secondary scales, morphology, new species, Seychelles, taxonomy

## Abstract

*Thaumatovalva* **gen. n.** is described and illustrated from the Afrotropical region. As currently defined the genus includes four species: *T. deprinsorum* **sp. n.** from the Democratic Republic of Congo; *T. albolineana* **sp. n.** (type species) from the Democratic Republic of Congo; *T. spinai* (Razowski & Trematerra), **comb. n.**, from Ethiopia and Nigeria; and *T. limbata* (Diakonoff), **comb. n.**, from the Seychelles and Kenya. *Thaumatovalva limbata* has been reared from the fruit of *Cordia somaliensis* Baker and *C. monoica* Roxb. (Boraginaceae) in Kenya. Although structures of the male and female genitalia are extremely similar among three of the four species, male secondary scales on the under surface of the hindwing easily distinguish them.

## Introduction

Male secondary sexual structures are abundant and diverse in the tortricid tribe Grapholitini (e.g., Komai 1999, Horak 2006). Examples include the modified antenna of *Gymnandrosoma aurantianum* Lima (e.g., Adamski and Brown 2001); the unusual scale brush on the metathorax of *Gymnandrosoma punctidiscanum* Dyar (e.g., Adamski and Brown 2001); the tibial hair pencils of *Cryptophlebia* and *Thaumatotibia* species (e.g., Diakonoff 1957, Komai 1999); the costal fold on the forewing of some species of *Dichrorampha* (Brown and Zacharides 2007); pockets in the hindwing bearing hairpencils and/or modified scales in some *Cydia* and *Thaumatotibia* species; and scale brushes (“coremata” *sensu* Komai 1999) surrounding the male genitalia in many species in the *Grapholita* group of genera (Komai 1999). Although the function of these structures is poorly studied, they are assumed to play a role in short-range courtship behavior (Baker and Carde 1979). As such, these features may function as pre-mating isolating mechanisms, and hence, may be of considerable taxonomic significance.

While working on the systematics and taxonomy of Afrotropical Grapholitini, we discovered specimens from the Belgian Congo that are superficially similar to *Thaumatotibia* and/or *Cryptophlebia* and have two conspicuously different patterns in the male secondary scaling on the underside of the hindwing. Upon dissection, it was revealed that males of both have genitalia nearly identical to those of *Thaumatotibia spinai* Razowski & Trematerra from Ethiopia, but also differ from that species in the male secondary scaling. A fourth presumed congener was discovered in material from Kenya, and it possesses genitalia identical to those of *Grapholita limbata* Diakonoff from the Seychelles. These four species differ from other species of *Thaumatotibia* in several diagnostic features, including the possession of a conspicuous dorsal subapical lobe on the phallus, large spindle-shaped secondary sex scales concealed in the last abdominal segment in the male, and a region of long slender scales along the anal region of the hindwing with the scales extending into a scale patch on the fifth abdominal segment in the male. The purpose of this contribution is to describe and illustrate the two new species and to propose a new genus to accommodate the four.

## Methods

**Map 1.**
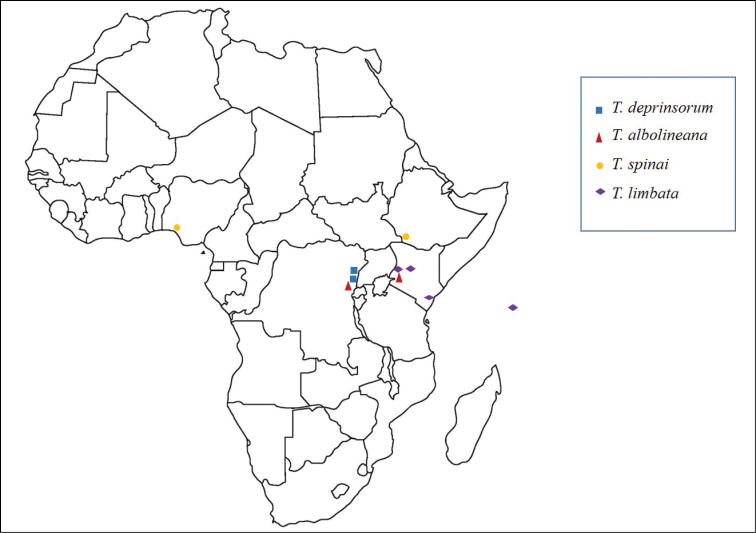
Geographic distribution of species of *Thaumatovalva*.

The specimens examined are from five sources: the collection of the Royal Museum for Central Africa, Tervuren, Belgium (RMCA) (n = 24); the collection of Pasquale Trematerra, Campobasso, Italy (PTC) (n = 1); the collection of the Natural History Museum, University of Oslo, Norway (NHMO) (n = 5); the collection of Todd Gilligan, Loveland, Colorado, USDA (TGC) (n = 1); and the collection of the National Museum of Natural History, Washington, DC, USA (USNM) (n = 14). Specimens not examined (*Thaumatovalva limbata*) are deposited in the Muséum National d’Historie Naturelle, Paris, France (MNHN) (n = 8); for these we relied on illustrations of the genitalia provided by Diakonoff (1969). Dissection methods followed those summarized by Brown and Powell (1991). Terminology for morphological structures follows Horak (1984, 2006). Forewing measurements include the fringe. Slide mounted genitalia were examined using dissecting and compound microscopes. Because adults of all species are superficially nearly indistinguishable except for male secondary scaling on the under surface of the hindwing, a description of the upper surface of the fore- and hindwing are given in the generic description to minimize redundancy in the species’ descriptions. Geographic distribution of the four species is shown in Map 1.

Nine specimens were sequenced for the 658-bp “barcode” region of the mitochondrial gene cytochrome oxidase I (COI) at the Biodiversity Institute of Ontario, University of Guelph using standard methods (Craft et al. 2010, Wilson 2012) and tissue from the leg of a dried adult moth. The nine specimens were selected based on their age (i.e., less than 10 years old). Unfortunately, most of the material available for examination was considerably older and resulted in no usable sequence data. A single sequence was obtained for *Thaumatotibia spinai* collected from the Ommo Valley in Ethiopia. Eight sequences were obtained for *Thaumatovalva limbata* collected from Kenya. Images captured of the pinned moths are stored on the BOLD (Barcode of Life Database) website.

## Results

### Thaumatovalva

Taxon classificationAnimaliaLepidopteraTortricidae

Timm & Brown gen. n.

http://zoobank.org/988181FE-30A9-43B0-8248-71FB16A3EB5C

#### Type species.

*Thaumatovalva albolineana* Timm & Brown, new species.

#### Diagnosis.

Superficially, species of *Thaumatovalva* are highly uniform in forewing pattern and very similar to pale specimens of *Thaumatotibia batrachopa* (Meyrick) or *Cryptophlebia rhynchias* (Meyrick). They are easily distinguished from those and all other species of *Thaumatotibia* and *Cryptophlebia* by the modified scaling of the under surface of the hindwing in the male, the patterns of which are species-specific. In addition, males of all species of *Thaumatovalva* possess a row of long slender scales along the anal region of the hindwing that extends into a modified patch of scales on abdominal segment V; highly unusual, elongate, spindle-shaped scale clusters concealed within the last abdominal segment; and variously developed, parallel, longitudinal rows of black scales subdorsally on abdominal segments 4–6 (occasionally weakly developed on 3). The tegumen of the male genitalia of *Thaumatovalva* is completely confluent with a membranous region behind it, forming what appears to be an extremely broadly, ovoid dorso-anterior portion of the tegumen. The phallus of all species of *Thaumatovalva* have a variably developed, somewhat digitate lobe from the dorsum in the distal 0.3. The latter two features appear to be unique within Grapholitini.

Komai (1999) identified five adult characters that support the monophyly of the clade *Thaumatotibia*+*Cryptophlebia*: (1) forewing with a blackish triangular pretornal patch; (2) forewing with accessory cell small or absent; (3) hindwing with short discal cell, especially in male; (4) T8 and sometimes preceding tergites in male with patch of long, easily removable mane-like scales; and (5) valva with a patch of very long, curled scales on the outer surface of the cucullus. Among these characters, *Thaumatovalva* lacks the pretornal patch and the mane-like scales.

The male genitalia of *Thaumatovalva*, with their many autapomorphies (e.g., the complex tegumen, the triangular process from the sacculus of the valva, the lobe-like process of the phallus), all serve to obscure the relationship of *Thaumatovalva* to its nearest relative. The simple, unmodified female genitalia, likewise, provide little compelling evidence of the position of the genus. An abundance of different types of male secondary scales is common to many Grapholitini, including *Cryptophlebia*, *Thaumatotibia*, *Talponia*, and others, however, these structures rarely provide compelling evidence of relationships. For example, males of *Multiquaestia* Karisch have a fascicle of slender scales extending from the base of the hindwing to the abdomen (Aarvik and Karisch 2009: figs 3–4). Although males of *Thaumatovalva* have a similar complex of scaling, the dense fascicle of scales present in *Multiquaestia* is quite different from that of *Thaumatovalva*. In *Thaumatovalva* the narrow, linear patch of sparse scales originates all along a membranous line in the anal region of the hindwing and inserts into a poorly defined pouch laterally on the abdomen. Barcode data place *Thaumatovalva* nearest two undescribed species of Grapholitini that lack compelling generic assignments. Hence, this gene is of little value in helping define the position of *Thaumatovalva* within Grapholitini.

#### Description.

**Figures 1–5.**
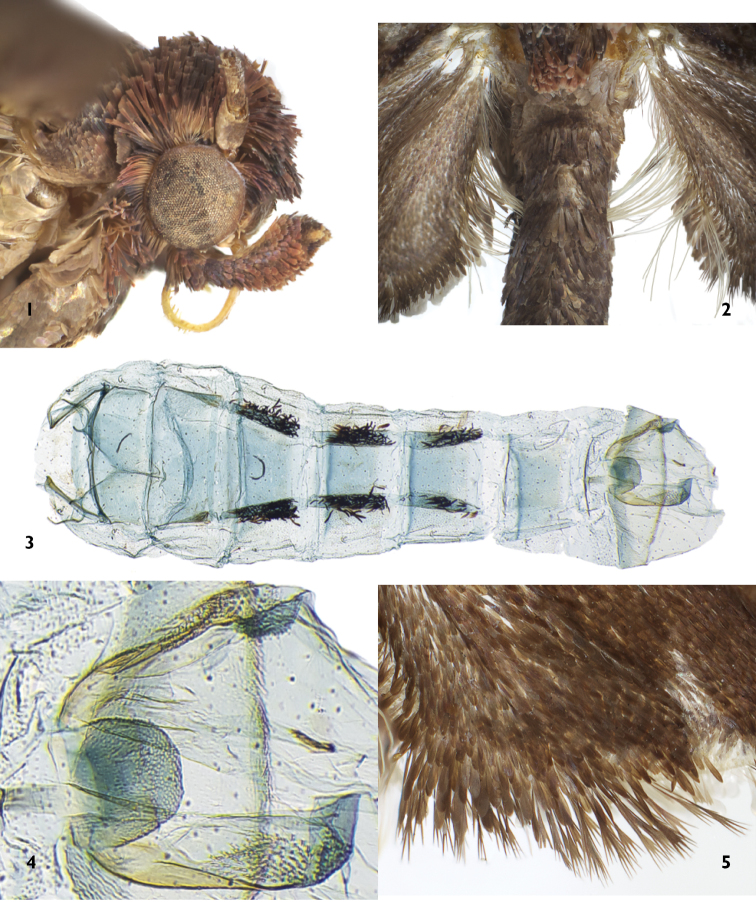
Morphological features of *Thaumatovalva*. **1** Head (lateral view) of *Thaumatovalva albolineana* **2** Male secondary scales extending from anal vein of hindwing into abdomen in *Thaumatovalva albolineana* **3** Paired patches of subdorsal secondary scales on denuded abdomen of male (USNM slide 144,506) **4** Sclerotized edge of segment VIII on denuded abdomen of male (USNM slide 144,506) **5** Forked scales along outer margin of hindwing.

**Head:** Vertex and upper frons rough scaled, scales mostly directly anterad, lower frons smooth scaled, with small appressed scales (Fig. 1); antenna ca. 0.5 length of forewing, weakly serrulate, scales in two rows per segment, sensory setae extremely short, inconspicuous in both sexes; ocellus moderately large, diameter ca. 0.5 that of base of scape; labial palpus upturned, third segment somewhat porrect, exposed, all segments combined ca. 1.2 times horizontal height of compound eye; proboscis present, presumably functional. **Thorax:** Tegula simple, unmodified; metathorax with upraised scale tuft [inconspicuous in worn specimens]; hindleg with slightly expanded scale tuft on tibia in male only. Forewing length 5.0–8.2 mm; forewing broad, length about 2.4 times width, slightly broaden distally, apex somewhat rounded; no costal fold or upraised scales; all veins separate, CuP well developed at margin, chorda weak, accessory cell weak; forewing dark brown, irregularly and faintly mixed with specks of charcoal, rust, and cream; inconspicuous pair of tiny cream dots ringed with orange near distal end of discal cell; narrow cream (faintly mixed with brown) irregular band extending along termen from apex to ca. 0.66 distance to tornus. Fringe pale brown. Forewing under surface nearly uniform brown, paler than uppersurface. Hindwing with all veins present, M_1_ and M_2_ widely separated, M_3_ and M_4_ short-stalked, cubital pecten weak in male, better developed in female; frenulum with one acanthus in male, three in female; male with long slender scales (ca. 30–40) along middle 0.7 of anal margin (Fig. 2) extending to narrow groove on abdominal segment V (groove not evident on dissected integumen), dissection revealing that hairs insert beneath anterior edge of dense, linear patch of black sex scales subdorsally on abdominal segment V (Fig. 3); underside of hindwing between anal angle and CuP with variously modified secondary scales in male (Figs 10–13); distally forked scales along outer edge of hindwing, most conspicuous in distal portion of anal margin (Fig. 5). **Abdomen:** Male with paired, parallel, thick, linear patches of black sex scales subdorsally on segments 4, 5, and 6 (easily lost during dissection) (Fig. 3); scales from anal margin of hindwing inserted into anterior edge of scale patch on segment V; dorsum of segment VIII of male with a narrow line of sclerotization and a weak stem from its middle; invaginated portion of segment VII of male with broad U-shaped sclerotized posterior edge bearing membranous lateral flaps to which a dense cluster (ca. 40–50) of highly modified, long, spindle-shaped male secondary scales are attached, and with rounded membranous mesal lobe (Fig. 4). Male genitalia (Figs 14–18) with uncus, socius, and gnathos absent. Tegumen broad, elongate-ovoid, confluent with membranous region behind it (anellus?), pedunculi not curved, vinculum small, U-shaped; valva narrow basally, broadening to middle, with (in *spinai*, *albolineana*, and *deprinsorum*) or without (in *limbata*) large, triangular expansion of valva along ventral edge just before cucullus, outer edge of cucullus evenly rounded, valva attenuate to acute, rounded apically; cucullus covered with fine hairline setae on outer surface; phallus narrow, relatively straight in basal portion, curved in distal portion, with bulbous subbasal lobe at point of articulation with phallus, with digitate subapical lobe of variable size; cornuti absent. Female genitalia (Figs 18–19) with papillae anales unmodified; apophyses long and slender, posteriores about same length anteriores; ventral portion of segment VII with a broad subrectangular sclerotized patch with V-shaped posterior edge and tiny ostium at apex of V, a short narrow trough from ostium to posterior edge of segment VIII; ductus bursae long, slender, frail, with ring-like sclerite near middle; corpus bursae pear-shaped, finely punctate (at high magnification), with two nearly equal size signa, each a long curved spine from a rounded sclerotized base.

#### Sexual dimorphism.

No apparent dimorphism in color or pattern, but females average slightly greater in forewing length and lack secondary scales on the under surface of hindwing, hind tibia, and abdomen.

#### Barcode data.

Eight (six reared from Kenya and two field-collected in Kenya) of the nine specimens have identical barcode data, and we assign them to *Thaumatovalva limbata*. All specimens resulted in standard 658-bp sequences. The reared specimens are considerably larger than the field-collected specimens, i.e., about the same size as *Thaumatovalva limbata* from the Seychelles, the type locality of the species. The ninth specimen, the holotype of *Thaumatotibia spinai*, shows about 1.0% divergence from the cluster of *Thaumatovalva limbata*.

#### Distribution and biology.

The genus is recorded from Ethiopia, Nigeria, the Democratic Republic of Congo, Kenya and the Seychelles (Map 1). Most species appear to occur in montane regions above ca. 1275 m, but *Thaumatovalva limbata* has been recorded from 35–1272 m; and a single specimen of *Thaumatotibia spinai* from Nigeria was collected at 54 m (Razowski and Wojtusiak 2012). Although the early stages are unknown, the larvae are assumed to be fruit feeders, as are most Grapholitini. *Thaumatovalva limbata* (cited as *Grapholita limbata* by Brown et al. 2014) has been reared from the fruit of *Cordia somaliensis* and *Cordia monoica* in Kenya.

#### Etymology.

The generic name is from the Latin *thaumato*, meaning “miracle” or “wonder,” and the morphological term “valva.”

#### Key to the males of *Thaumatovalva*

**Table:** 

1	Upper surface of hindwing pale gray-brown; under surface of hindwing without white sex scales along anal margin or inner angle of wing	*Thaumatovalva limbata*
1’	Upper surface of hindwing nearly uniform brown; under surface of hindwing with white sex scales along anal margin or inner angle of wing	2
2	Undersurface of hindwing with broad area of white scales along anal region	*Thaumatovalva deprinsorum*
2’	Undersurface of hindwing with narrow line of white scales along margin from lower edge of anal margin to about CuP	3
3	White marginal scales short and compact (i.e., about 3–5 times as long as wide)	*Thaumatovalva albolineana*
3’	White marginal scales longer (i.e., about 7–10 times as long as wide)	*Thaumatovalva spinai*

### Thaumatovalva deprinsorum

Taxon classificationAnimaliaLepidopteraTortricidae

Timm & Brown sp. n.

http://zoobank.org/1B943CE5-BA8C-4407-A8AE-32AF21EF252A

**Figures 6–9.**
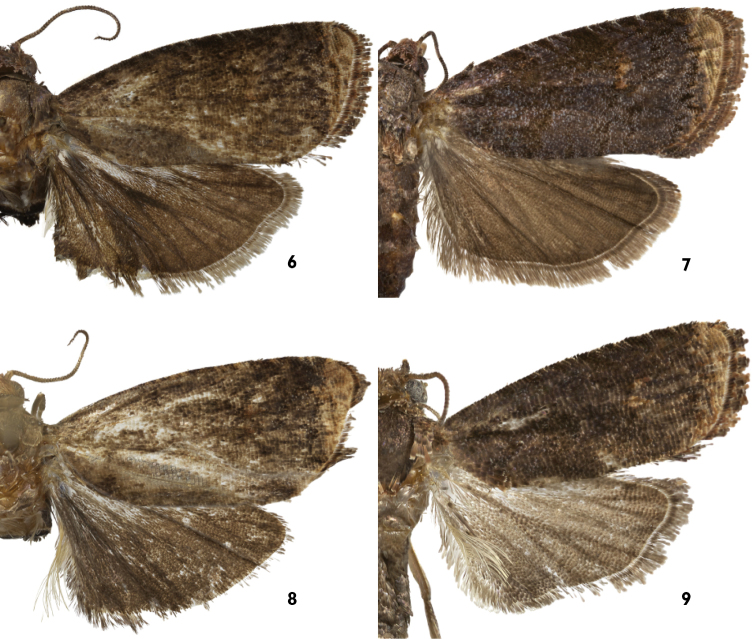
Upper suface of wings of *Thaumatovalva*. **6** *Thaumatovalva deprinsorum* **7** *Thaumatovalva albolineana* **8** *Thaumatotibia spinai* [image enhanced using best parts of both forewings] **9** *Thaumatovalva limbata*.

**Figures 10–13.**
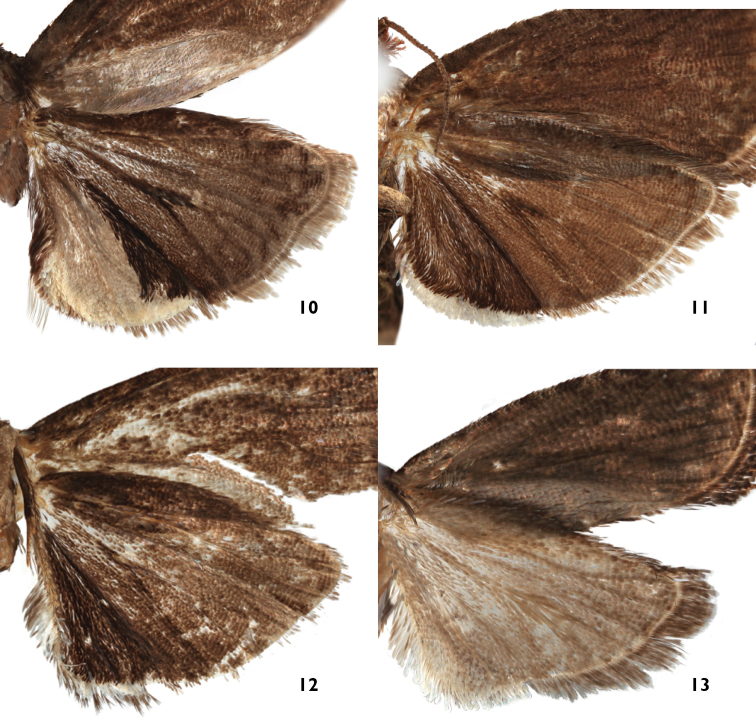
Under surface of the hindwing of male *Thaumatovalva*. **10** *Thaumatovalva deprinsorum* **11** *Thaumatovalva albolineana* **12** *Thaumatotibia spinai* **13** *Thaumatovalva limbata*.

**Figures 14–19.**
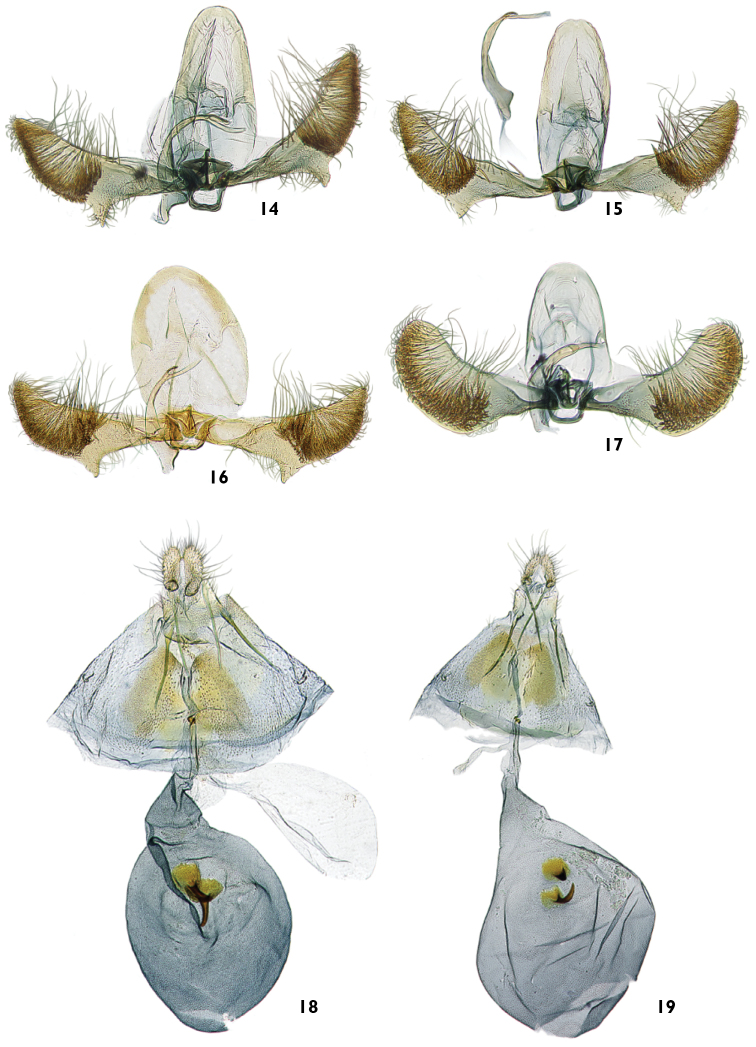
Male and female genitalia of *Thaumatovalva*. **14** Male of *Thaumatovalva deprinsorum* (USNM slide 144,491) **15** Male of *Thaumatovalva albolineana* (USNM slide 144,488) **16** Male of *Thaumatotibia spinai* (slide 2656) **17** Male of *Thaumatovalva limbata* (USNM slide 144,490) **18** Female of *Thaumatovalva deprinsorum* (USNM slide 144,492) **19** Female of *Thaumatovalva albolineana* (USNM slide 144,489).

Figs 6 , 10 , 14 , 18

#### Type material.

Holotype ♂, Democratic Republic of Congo, P. N. A., Secteur Nord, Mutsora, 1200 m, 4 Dec 1957, A. Vanschuytbroeck (RMCA). Paratypes (12♂, 1♀). Democratic Republic of Congo: P. N. A.: Secteur Nord, Bumali village pres Mutwanga, 1300 m, piege lumineux, 28 Nov 1957 (3♂), A. Vanschuytbroeck (RMCA). Secteur Nord, Mutsora, 1200 m, 4 Dec 1957 (1♂), A. Vanschuytbroeck (RMCA). Secteur Nord, riv. Talya, affl. dr. Lume, 1260 m, 26 Sep 1956 (1♂), A. Vanschuytbroeck (RMCA). Massif Ruwenzori, riv. Kakalari, 1800 m, affl. Bombi, 28 Nov 1957 (5♂, 1♀), A. Vanschuytbroeck (RMCA). Massif Ruwenzori, Kyandolire (lieu-dit), 1810 m (sous bananiers savages), 24 Dec 1957 (1♂), A. Vanschuytbroeck (RMCA). Massif Ruwenzori, Gotte Ibatama, 1610 m (lumiere), 4 Ma00y 1958 (1♂), A. Vanschuytbroeck (RMCA).

#### Diagnosis.

*Thaumatovalva deprinsorum* is nearly indistinguishable from *Thaumatovalva albolineana* and *Thaumatotibia spinai* in forewing pattern and size, but it is easily separated by the modified scaling on the underside of the hindwing. In *Thaumatovalva deprinsorum* the patch of white scales occupies the entire inner portion of the anal region with a short dash extending beyond the patch along the wing margin (Fig. 10).

#### Description.

***Male*.** **Head:** Vertex dark brown mixed with lighter brown, upper frons concolorous with vertex, lower frons cream; labial palpus slightly lighter than vertex, inner surface slightly lighter yet; antennal scaling brown. **Thorax:** Dorsum dark brown, mixed with lighter brown, tegula concolorous with dorsum; hind tibia with dark brown patch of expanded scales. Forewing length 6.5–8.2 mm (mean = 7.0; n = 10), as described for genus; hindwing upper surface nearly uniformly brown, under surface concolorous with forewing undersurface with white scaling from anal margin to about CuP, extending through anal region from wing margin to approximately CuP (Fig. 10). **Abdomen:** Brown; black scale patches not visible on undissected specimens. Genitalia (Fig. 14) with valva narrow basally, broadening to middle, with large, triangular expansion of valva ventrally just before cucullus, outer edge of cucullus rounded, valva attenuate through cucullus, apex rounded; phallus narrow, with bulbous subbasal lobe and conspicuous elongate dorsal lobe ca. 0.66 distance from base to tip.

***Female*.** **Head and thorax:** Essentially as described for male, except forewing length 8.0 mm (n = 1). **Abdomen:** Brown. Genitalia (Fig. 18) essentially as described for genus; ventral portion of segment VIII with a broad subrectangular semi-sclerotized patch with V-shaped posterior edge, ostium situated at end of short narrow trough extending from vertex of V-shaped sclerotized region; ductus bursae long, ca. twice length of corpus bursae, slender, frail, with ring-like sclerite near middle; ductus seminalis originating ca. 0.5 distance between sclerotized ring and corpus bursae; ductus gradually broadening into corpus in distal 0.1; corpus bursae pear-shaped, finely punctate (at high magnification), with two nearly equal signa, each a long curved spine from a rounded sclerotized base.

#### Distribution and biology.

This species is known only from the middle elevations (1200–1810 m) of the Democratic Republic of Congo. It has been collected primarily in November and December, with a single record from May.

#### Etymology.

The specific epithet is a patronym for Willy and Jurate De Prins.

### Thaumatovalva albolineana

Taxon classificationAnimaliaLepidopteraTortricidae

Timm & Brown sp. n.

http://zoobank.org/290DE30B-553D-4DC4-B014-4F6DC9F1AE6F

Figs 1 , 2 , 7 , 11 , 15 , 19

#### Type material.

Holotype ♂, Democratic Republic of Congo, North Kivu, Rutshuru, [1.18°S, 29.45°E] [1275 m], Jun 1937, J. Ghesquiere (RMCA). Paratypes (5♂, 5♀). Democratic Republic of Congo: North Kivu: Rutshuru, Feb 1937 (1♂, 1♀), Mar 1937 (2♀), Apr 1937 (2♂), May 1937 (1♀), Jun 1937 (2♂, 1♀), J. Ghesquiere (RMCA). Kenya: Kakamega District: Kakamega Forest reserve, Rondo Retreat Centre, 1598 m, 00°13’37.9”N, 34°53’04.6”E, 23–26 Nov 2010 (1♂), T. Gilligan & A. Mukiri (TGC).

#### Diagnosis.

*Thaumatovalva albolineana* is similar to *Thaumatovalva deprinsorum* and *Thaumatotibia spinai* both superficially and in genital morphology. However, males of *Thaumatovalva albolineana* are easily distinguished by the modified scaling on the under surface of the hindwing, which consists of a narrow line of short, compact white scales along the rounded portion of the wing from the end of the anal region to about vein CuP.

#### Description.

***Male*.** **Head:** Vertex dark brown mixed with lighter brown, upper frons concolorous with vertex, lower frons cream; labial palpus slightly lighter than vertex, inner surface slightly lighter yet; antennal scaling brown. **Thorax:** Dorsum dark brown, mixed with lighter brown, tegula concolorous with dorsum; hind tibia with dark brown patch of expanded scales. Forewing length 5.8–7.5 mm (mean = 6.5; n = 5); forewing as described for genus; hindwing upper surface nearly uniformly brown, under surface concolorous with forewing under surface, but slightly darker in anal region; a conspicuous narrow band of shiny, pearly-white scales along wing margin, extending from lower edge of anal margin to approximately CuP (Fig. 11). **Abdomen:** Brown (black scale patches not visible on undissected specimens). Genitalia (Fig. 15) with valva narrow basally, broadening to middle, with large, triangular expansion of valva ventrally just before cucullus, outer edge of cucullus rounded, valva attenuate through cucullus, apex rounded; phallus narrow, with bulbous subbasal lobe and conspicuous elongate dorsal lobe ca. 0.66 distance from base to tip.

***Female*.** **Head and thorax:** Essentially as described for male, except forewing length 6.0–8.0 mm (mean = 7.4; n = 5). **Abdomen:** Brown. Genitalia (Fig. 19) essentially as described for genus; ventral portion of segment VIII with a broad subrectangular semi-sclerotized patch with V-shaped posterior edge, ostium situated at end of short narrow trough extending from vertex of V-shaped sclerotized region; ductus bursae long, ca. twice length of corpus bursae, slender, frail, with ring-like sclerite near middle; ductus seminalis originating ca. 0.5 distance between sclerotized ring and corpus bursae; ductus gradually broadening into corpus in distal 0.1; corpus bursae pear-shaped, finely punctate (at high magnification), with two nearly equal sized signa, each a long curved spine from a rounded sclerotized base.

#### Distribution and biology.

*Thaumatovalva albolineana* is known nearly exclusively from the type locality of Rutshuru in the Democratic Republic of Congo, but there is a single specimen from Kenya. Specimens have been collected between about 1500 and 1600 m elevation. Adults have been collected primary from February through June, with a single record from November (Kenya). Nothing is known of the early stages.

Etymology. The specific epithet refers to the narrow band of white scales on the underside of the hindwing.

### Thaumatovalva spinai

Taxon classificationAnimaliaLepidopteraTortricidae

(Razowski & Trematerra) comb. n.

Figs 8 , 12 , 16

Thaumatotibia spinai Razowski & Trematerra, 2010: 65.

#### Type material.

Holotype ♂, Ethiopia, Omo Valley, Dowro Zone, Tarcha, 1400 m, 16 Apr 2004, A. Sciarretta & G. Spina (PTC). Paratypes (1♂). Ethiopia: Omo Valley, Dowro Zone, Tarcha, 1400 m, 16 Apr 2004, A. Sciarretta & G. Spina (PTC).

#### Diagnosis.

*Thaumatovalva spinai* is nearly indistinguishable from *Thaumatovalva albolineana* and *Thaumatovalva deprinsorum* in forewing pattern and size, but it can be separated by the modified scaling on the underside of the hindwing. In *Thaumatotibia spinai* the white scales along the inner margin of the hindwing are long and slender and dark-tipped compared to the short, stout, completely white scales of *Thaumatovalva albolineana*.

#### Description.

***Male*.** **Head:** Vertex dark brown mixed with lighter brown, upper frons concolorous with vertex, lower frons cream; labial palpus slightly lighter than vertex, inner surface slightly lighter; antennal scaling brown. **Thorax:** Dorsum dark brown, mixed with lighter brown, tegula concolorous with dorsum; hind tibia with dark brown patch of expanded scales. Forewing (Fig. 8) length 6.0 mm (n = 2); forewing as described for genus; hindwing upper surface nearly uniformly brown, undersurface slightly darker in anal region; a conspicuous band of shiny white scales along margin of wing extend from lower edge of anal margin to approximately CuP (Fig. 12). **Abdomen:** Brown; black scale patches not visible on undissected specimens. Genitalia (Fig. 16) with valva narrow basally, broadening to middle, with large, triangular expansion of valva ventrally just before cucullus, outer edge of cucullus rounded, valva attenuate through cucullus, apex rounded; phallus narrow, with bulbous subbasal lobe and conspicuous elongate dorsal lobe ca. 0.66 distance from base to tip.

***Female*.** Unknown.

#### Distribution and biology.

*Thaumatovalva spinai* is known only from the type locality in Ethiopia at 1400 m elevation. Razowski and Wojtusiak (2012) report a single male from Nigeria (Bendel State, Okomu Forest, 20 Oct 1985), but we are uncertain whether it is conspecific with the holotype. The early stages are unknown.

#### Etymology.

The species was named for one of the collectors of the holotype, Giuesppe Spina.

#### Remarks.

The holotype male and paratype of this species are both in poor condition, so comparison of forewing pattern is limited. The image of the adult (Fig. 11) is based a photo reconstruction of the holotype, using the best parts of both forewings. An unmodified image of the holotype can be found in Razowski and Trematerra (2010).

### Thaumatovalva limbata

Taxon classificationAnimaliaLepidopteraTortricidae

(Dakonoff, 1969) comb. n.

Figs 9 , 13 , 17

Grapholita limbata Dakonoff, 1969: 85; Brown 2005: 361; Brown et al. 2014: in press.

#### Type material.

Holotype ♂, Seychelles, Praslin Island, 27 May 1960, M. Gerber (MNHN). Paratypes (4♂, 3♀). Seychelles: Mahé Island, Beau Vallon, 27 Apr 1959 (1♀), 29 Mar 1960 (1♂), 19 Mar 1959 (1♂, 1♀), 27 May 1959 (1♂), H. Legrand (MNHN). Cosmoledo Island 16 Sep 1959 (1♀), H. Legrand, 19 Oct 1959 (1♂), M. Gerber (MNHN).

#### Additional specimens examined.

KENYA: 18 km SW Malindi, Watamu, 35 m, 3°22'S, 39°59'E, 15 Mar 2004 (3♂), J. & W. De Prins (RMAC). Coast Province, Sabaki, 10 m, 3°09.28'S, 40°08.05'E, 10 Jul 2001 (2♂, 3♀), reared from fruit of *Cordia somaliensis* Baker (Boraginaceae), R. Copeland (USNM). Rift Valley Province, Mathews Range, 1272 m, 1°10.827'N, 37°17.876'E, 18 Jan 2004 (4♂, 4♀), reared from fruit of *Cordia monoica* Roxb. (Boraginaceae), R. Copeland (USNM). Rift Valley Province, Masai Lodge, 1670 m, 37MBU 5679 4682, 25 Nov 2010 (2♂, 2♀), L. Aarvik & D. Agassiz (NHMO). Rift Valley Province, Mount Elgon National Park, Chorlin Gate, Rongai Camp, 2206 m, 17-21 Nov 2006 (1♀), L. Hansen & K. Sund (NHMO). SEYCHELLES: Aldabra Atoll, Ile Picard Settlement, 12–22 Mar 1986 (1♀), D. Adamski (USNM).

#### Diagnosis.

*Thaumatovalva limbata* shares a similar forewing pattern with its congeners, but males average slightly smaller in forewing length (5.5 mm vs. 7.0 mm), it also has a paler gray brown rather than dark brown under surface of the hindwing, and its male genitalia lack the characteristic triangular process from the venter of the valva. The patch of modified sex scales concealed in the distal end of the abdomen consists of only four large sausage-shaped structures, two on each side, rather than 40–50 scales present in congeners, and the hindwing has no conspicuous white scaling on the under surface.

*Thaumatovalva limbata* is superficially similar to “*Eucosma*” *chloroterma* Meyrick, 1913, described from Pretoria, South Africa, but the male genitalia of the latter (see Clarke 1958: 355) have little in common with those of *Thaumatovalva limbata*.

#### Description.

***Male*.** **Head:** Vertex dark brown mixed with lighter brown, upper frons concolorous with vertex, lower frons cream; labial light brown than vertex, inner surface cream; antennal scaling brown. **Thorax:** Dorsum dark brown, mixed with lighter brown, tegula dark brown mixed with lighter brown, most scale brown with cream tip; hind tibia with dark brown with expanded scales poorly developed. Forewing length 5.0–6.0 mm (mean = 5.5; n = 5); forewing as described for genus; hindwing nearly uniform grayish brown, conspicuously paler than forewing; patch of ca. 30 slender scales from anal margin of hindwing not inserted into scales of abdomen. Fringe conolorous with hindwing. Hindwing under surface concolorous with forewing undersurface, paler than in congeners; an inconspicuous narrow row of slightly enlarged brown scales along margin of wing from lower edge of anal margin to approximately CuP (Fig. 13). **Abdomen:** Brown; black scale patches subdorsally on abdominal segments 3–5 weakly developed (not visible in undissected specimens); sclerotized posterior edge of segment VIII bearing four (two on each side), long, slender sausage-shaped scales. Genitalia (Fig. 17) with valva narrow basally, broadening to middle, without triangular expansion before cucullus, outer edge of cucullus rounded, valva attenuate through cucullus, apex conspicuously more rounded than in congeners; phallus narrow, with bulbous subbasal lobe and small rounded dorsal lobe ca. 0.5 distance from base to tip, distal 0.2 beyond lobe slight curved.

***Female*.** **Head and thorax:** As described for male, except forewing length 6.0-8.0 mm (mean = 6.8; n = 6). **Abdomen.** Genitalia as described for the genus and figured for congeners.

#### Distribution and biology.

*Thaumatovalva limbata* is known from Praslin, Cosmoledo, and Mahé islands in the Seychelles, and from Kenya on the mainland. Adults have been reared from larvae collected in the fruit of *Cordia somaliensis* and *Cordia monoica* in Kenya, where the species occupies a broad elevational range from 10 to 2206 m.

#### Remarks.

Based on forewing size, small specimens from Kenya (n = 7, from Watamu and Masai Lodge) appear to represent an undescribed species. However, the genitalia of these small specimens are identical to those of larger specimens of *Thaumatovalva limbata* from the Seychelles (see Diakonoff 1969: fig. 11, holotype) and reared specimens from Kenya. Furthermore, DNA barcodes do not separate the small specimens from the larger Kenyan specimens, the latter of which are identical to *Thaumatovalva limbata* from the Seychelles. All eight of our *Thaumatovalva limbata* sequences are identical throughout their sequenced length. Nonetheless, the broad elevational range and size differences suggest that more than one species may be concealed under this name.

## Supplementary Material

XML Treatment for Thaumatovalva

XML Treatment for Thaumatovalva deprinsorum

XML Treatment for Thaumatovalva albolineana

XML Treatment for Thaumatovalva spinai

XML Treatment for Thaumatovalva limbata
